# Circadian influences on central nervous system barriers and the glymphatic system

**DOI:** 10.3389/fphys.2025.1622236

**Published:** 2025-06-23

**Authors:** Brittany D. Elliott, Claire O. Kisamore, Randy J. Nelson, A. Courtney DeVries, William H. Walker

**Affiliations:** ^1^ Department of Neuroscience, Rockefeller Neuroscience Institute, Morgantown, WV, United States; ^2^ West Virginia University Cancer Institute, Morgantown, WV, United States; ^3^ Department of Medical Oncology, West Virginia University, Morgantown, WV, United States

**Keywords:** circadian rhythms, blood-brain barrier, blood-cerebrospinal fluid barrier, glymphatic system, CNS barriers

## Abstract

The central nervous system (CNS), comprising the brain and spinal cord, is fortified by complex barriers that protect the underlying organs and maintain homeostasis. The importance of proper fortification and homeostatic regulation provided by these systems has broad implications for many physiological processes and several pathological conditions are associated with their disruption. Recent studies support the notion that CNS barriers and fluids are regulated by circadian rhythms. Whereas reciprocal associations between the structural and functional integrity of neural barriers and disease states are well-established, the role of circadian rhythms in mediating these relationships remains unspecified. The goals of this review are to provide a general overview of three primary systems responsible for maintaining CNS homeostasis, namely the blood-brain barrier, blood-cerebrospinal fluid barrier, and glymphatic system, and to synthesize recent evidence highlighting the role of circadian rhythms as a critical regulator of CNS fluid and barrier function.

## Graphical Abstract

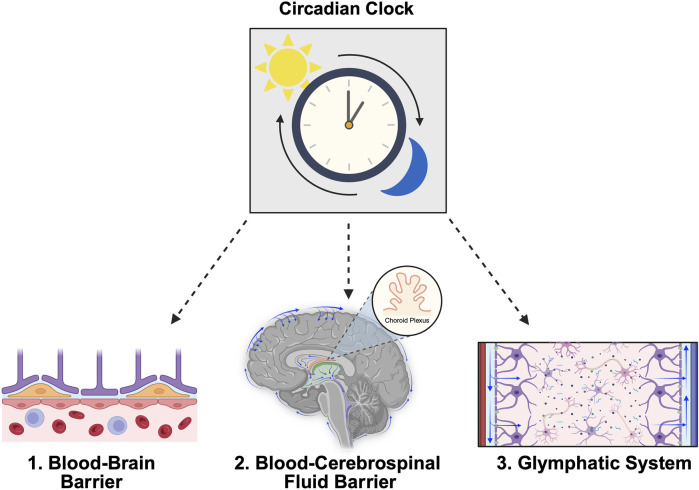

## 1 Introduction

The central nervous system (CNS), consisting of the brain and spinal cord, is regulated by several complex structures and systems referred to as CNS barriers. In healthy individuals, these systems are responsible for protecting the CNS, relaying information between the brain and periphery, and maintaining neural homeostasis. Similar to the peripheral processes of the endocrine, cardiovascular, digestive, immune, and metabolic systems, CNS barrier and fluid functions are also influenced by circadian rhythms (Patke et al., 2020; Steffensen et al., 2023; Fame et al., 2023; Edelbo et al., 2023; Vizcarra et al., 2024). Further, the dysfunction of CNS barriers is implicated in several pathological conditions. Although the bidirectional relationship between pathology and CNS barrier dysfunction is well-established for many conditions, the roles of barrier-specific circadian mechanisms in disease etiology and progression have not been previously described. Given the emergent interest in leveraging the circadian biology of CNS barriers for therapeutic optimization, there is an essential need to understand how circadian components uniquely influence the properties of each barrier under both healthy and pathological conditions.

## 2 Circadian rhythms overview

**FIGURE 1:**
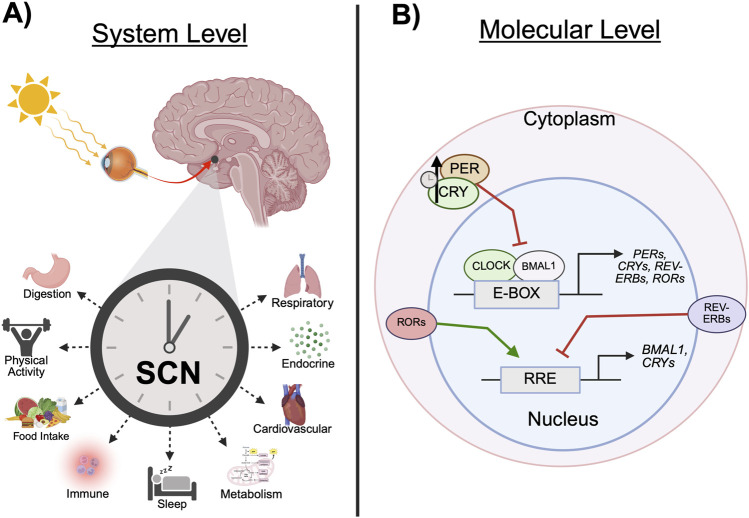
Circadian clock mechanisms. **(A)** Light enters the retina and travels along the retinohypothalamic tract to the SCN. Temporal information projected from the SCN allows for the synchronization of peripheral behavioral and physiological rhythms. **(B)** The expression of core clock genes, including *CRYs*, *PERs, REV-ERBs,* and *RORs*, is driven by the CLOCK:BMAL1 heterodimer, which binds to E-boxes in the promotor region of the gene. CRY and PER heterodimers inhibit the transcription of its constituent genes via CLOCK:BMAL1 repression. Over time, CRY:PER is degraded and the inhibition of CLOCK:BMAL1 is released, allowing transcription to resume. This process is referred to as the transcription-translation feedback loop. In a parallel mechanism, REV-ERBα and *ROR*α bind to RREs to inhibit or drive clock gene expression, respectively. (BMAL1, brain and muscle ARNT-like protein 1; CLOCK, circadian locomotor output cycle kaput; CRY, cryptochrome; E-box, enhancer box regulatory element; PER, period; SCN, suprachiasmatic nucleus; REV-ERB, nuclear receptor subfamily 1 group D member 1; ROR, retinoic acid receptor-related orphan receptor; RRE, rev response element. Schematic created with BioRender.).

Circadian rhythms are biological phenomena present in all domains of life that influence nearly every aspect of physiology and behavior (Bumgarner and Nelson, 2021). Derived from the Latin expression *circa diem*, meaning “about a day,” circadian rhythms exhibit oscillations approximately every 24 h (Patke et al., 2020). Entrainment cues from the environment, referred to as *zeitgebers* (time givers in German), inform the timing of these processes and allow organisms to optimize energy intake and expenditure to optimally meet demands across the solar day. Although physical activity, caloric intake, and temperature are important *zeitgebers*, the most predominant is the daily light-dark cycle. Light activates intrinsically photosensitive retinal ganglion cells within the eye, which transmit photic information along the retinohypothalamic tract (RHT) to the suprachiasmatic nuclei (SCN) within the anterior hypothalamus (Colwell, 2011). The SCN then projects to various regions within the thalamus and hypothalamus and allow for the intrinsic synchronization of system, organ, cellular, and genetic rhythms (Figure 1A) (Starnes and Jones, 2023).

The SCN can be partitioned into two structural components: a dorsal shell containing arginine vasopressin (AVP) neurons and the ventral core, consisting of photosensitive vasoactive intestinal polypeptide (VIP) and gastrin-releasing peptide neurons (Welsh et al., 2010). Neuronal activity in the SCN core is greatest during the day in both diurnal and nocturnal species, whereas nighttime firing is almost entirely absent (Colwell, 2011). Mechanistically, photic information delivered via RHT innervation results in the release of glutamate and pituitary adenylate cyclase-activating peptide (PACAP). The activation of N-methyl D-aspartate receptors and voltage-gated calcium channels by glutamate results in an increase of intracellular calcium and the downstream activation of calcium-dependent protein kinases (CAMK). Similarly, PACAP receptor 1 activation upregulates intracellular cyclic AMP (cAMP; cyclic adenosine 3′,5′-cyclic monophosphate), which then activates protein kinase A (PKA). Together, PKA and CAMK phosphorylate cAMP response element (CRE)-binding protein (CREB). This process, referred to as activation of the cAMP-PKA-CREB signaling pathway, then temporally initiates the transcription of circadian clock genes and promotes cell-autonomous synchronization to the environment (Starnes and Jones, 2023).

On the molecular level, the expression of clock genes is driven by two cytoplasmic proteins, circadian locomotor output cycle kaput (CLOCK) and brain and muscle ARNT-like protein 1 (BMAL1), which dimerize and translocate to the nucleus. There, the CLOCK:BMAL1 heterodimer regulates transcription by binding to enhancer box regulatory elements (E-boxes) at the promoter region of the clock genes, such as those in the *Cryptochrome* (*Cry*) and *Period* (*Per*) families (Cox and Takahashi, 2019). CRY and PER proteins dimerize and accumulate in the cytoplasm before translocating back to the nucleus where the heterodimer inhibits the transcription of its constituent genes via CLOCK:BMAL1 repression. Over approximately 24 h, the increasing accumulation of CRY and PER proteins become ubiquitinated and degraded, thus releasing the inhibition of CLOCK:BMAL1 and reinitiating transcription (Figure 1B). In a parallel mechanism, the CLOCK:BMAL1 dimer drives the expression of nuclear receptors subfamily 1 group D member 1 (NR1D1*/*REV-ERBα) and nuclear receptor subfamily 1 group D member 2 (NR1D2/REV-ERBβ). REV-ERBs compete with retinoic acid receptor-related orphan receptors (RORs) to bind to rev response elements (RREs) on the promotor region of *Bmal1* and, respectively, repress or drive expression (Cox and Takahashi, 2019). The CLOCK/BMAL1 and REV-ERB/ROR loops regulate the transcription of additional genes, *Nfil3* and *Dpb,* which comprise a third feedback loop. In brief, NFIL3:DPB dimers bind to D-boxes and drive the transcription of several clock genes (Cox and Takahashi, 2019). Together, the processes underlying these three loops comprise the intrinsic molecular clock. For an in depth description of photic entrainment of the circadian system, refer to the 2021 review by Ashton et al. (2022).

## 3 Circadian rhythms and the blood-brain barrier

### 3.1 Overview of the blood-brain barrier

**FIGURE 2:**
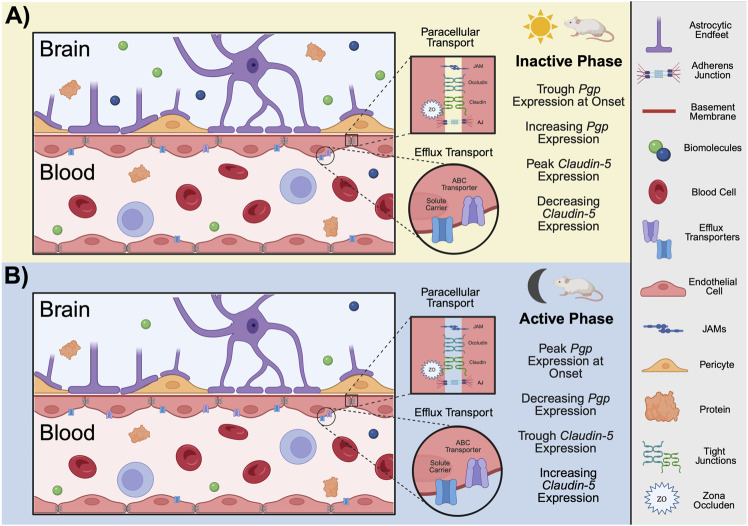
Function and structure of the BBB in rodents. The BBB is composed of a monolayer of endothelial cells known as BMECs. Surrounding the ECs of neural blood vessels are basement membrane proteins, pericytes, and astrocytic endfeet, which together comprise the NVU. BMECs are separated junction complexes including JAMs, TJs, and AJs which restrict paracellular transport. In mice, efflux pumps, such as ABC transporters and SLCs, are located on both the luminal and abluminal membranes of the BBB. At the onset of the **(A)** inactive phase for mice, *PgP* expression is at its trough but gradually increases across the light period. On the contrast, the expression of *Claudin-5,* a TJ transporter, peaks at the onset of inactivity but decreases with time. At the onset of the **(B)** active phase for mice, *Claudin-5* expression is at its trough and gradually increases with time. *Pgp* expression peaks at the onset of the active phase and decreases throughout the night. Conclusions regarding period-specific permeability cannot be drawn for several reasons: 1) time between peak expression and peak activity likely varies, 2) time of peak activity/expression likely varies between different transport mechanisms (paracellular *versus* efflux), as observed here, and 3) it is unknown if the expression/activity of mechanistically similar transport proteins (e.g., PgP *versus* BCRP) peak at similar times. Given these uncertainties even within species, only conclusions drawn from studies involving mice are demonstrated here. (ABC, ATP-binding cassette; AJs, adherens junctions; BCRP, breast cancer resistance protein; BMECs, brain microvascular endothelial cells; ECs, endothelial cells; JAMs, junctional adhesion molecules; Pgp, p-glycoprotein; SLCs, solute carriers; TJs, tight junctions; NVU, neurovascular unit; ZO, zonula occluden. Schematic created with BioRender.).

The blood-brain barrier (BBB; Figure 2) consists of a vascular network throughout the CNS composed of a thin monolayer of endothelial cells (ECs) known as brain microvascular endothelial cells (BMECs). The main functions of the BBB are to provide the brain parenchyma with key nutrients while keeping xenobiotic substances (e.g., drugs, bacteria, *etc.*) from infiltrating the surrounding tissue (Daneman and Prat, 2015). The distance between the intravascular (luminal) surface and the brain parenchyma surrounding the extravascular surface (abluminal) is ∼300–500 nm in human brain microvessels (Cornford and Hyman, 2005). These cells are separated by unique tight junctions (TJs) that deter a large majority of paracellular transport (Daneman and Prat, 2015). Directly surrounding the ECs of neural blood vessels are basement membrane proteins, pericytes, and astrocytic endfeet, which together comprise the neurovascular unit (NVU) (Kadry et al., 2020). These cell types also influence overall permeability of the BBB (Daneman and Prat, 2015). For example, pericytes and astrocytic endfeet signal to BMECs to regulate blood flow and defend against apoptosis (Ramsauer et al., 2002), among other functions (Kadry et al., 2020; Abbott et al., 2010).

BMECs are highly specialized and have unique characteristics compared to peripheral ECs. They are non-fenestrated and harbor multiple transport systems which contribute to the incredible selectivity of the BBB (Kadry et al., 2020). Among the many transport systems present at the BBB, one of the most clinically relevant is efflux by ATP-binding cassette (ABC) transporters. ABC transporters, also referred to as efflux pumps, are transcellular proteins that use ATP to transport solutes against their concentration gradients (Demeule et al., 2002; Mahringer and Fricker, 2016). One function of these proteins is to direct xenobiotic substances away from the brain parenchyma by actively expelling them back into the vasculature. P-glycoprotein (Pgp) is arguably the most studied ABC transporter due to its ubiquity throughout the body and major implications for drug delivery (Demeule et al., 2002). Substrates for Pgp span a plethora of pharmaceutics including some chemotherapies, antibiotics, and opioids (Conrad et al., 2024; Karthika et al., 2020; Sharom, 2011). The expression of Pgp along with other major ABC transporters, such as breast cancer resistance protein (BCRP) and multi-drug resistance proteins (MRPs), present a significant hurdle in pharmaceutical delivery to the brain. Researchers have invested decades in overcoming this challenge through innovative solutions from first-generation strategies (e.g., liposomal formulations) to more modern approaches like nanoparticles and biomimetic systems (Wu et al., 2023). However, the lack of consistency in ABC transporter expression across species complicates this research. For instance, Pgp is expressed at relatively different levels in humans, mice, and rats (Zhang et al., 2023; Uchida et al., 2011). Furthermore, Pgp is expressed on both the luminal and abluminal surfaces of BMECs in humans, but only on the luminal surface of the BBB in mice (Morris et al., 2017). BCRP, which is present at the luminal surface in humans and rodents, is expressed in higher levels in humans than in mice and rats (Morris et al., 2017). Although Pgp humanized mice are currently used to study efflux mechanisms at the BBB (Krohn et al., 2018), this model fails to capture the species-specific dynamic processes of ABC transporters working together in concert to protect the brain parenchyma. For example, the inhibition of one results in a compensatory mechanism among ABC transporters (Cisternino et al., 2004). Indeed, knocking out Pgp in mice has been demonstrated to increase expression of BCRP at the BBB (Cisternino et al., 2004). Thus, studies of drug efflux and delivery at the BBB are complex and results often vary.

Similar to ABC transporters, solute carriers (SLCs) are dispersed across both the luminal and abluminal surfaces of BMECs (Kadry et al., 2020). SLCs are transmembrane channels that function to transport specific vital nutrients that cannot diffuse transcellularly due to size or charge. This includes glucose, amino acids, hormones, anions, vitamins, carbohydrates, and many more (Kadry et al., 2020; Hu et al., 2020). These channels function primarily through passive diffusion, and some utilize ion concentration gradients (e.g., Na^+^ and K^+^) to drive the transport of molecules such as glutamate (Hu et al., 2020; Takahashi et al., 2015). Additionally, substances can cross the BBB via vesicular transport. This involves either adsorptive-mediated or receptor-mediated transcytosis of lipophilic vesicles containing materials that are too large to otherwise breach the BBB (Kadry et al., 2020). ECs have a specific intracellular vesicular trafficking mechanism that allows for the avoidance of lysosomes during transcytosis through BMECs (Kadry et al., 2020; Foroozandeh and Aziz, 2018). This is accomplished through endocytosis at a caveola (Latin, “little cave”), an invagination on ECs. BMECs have fewer caveolae than some peripheral ECs, as well as fewer overall intracellular endocytic vesicles (Kadry et al., 2020).

Arguably one of the most crucial defining features of the BBB is the expression of TJs and adherens junctions (AJs). These junctional complexes are responsible for the strict tethering of endothelial cells to ensure that the brain vasculature does not allow for paracellular transport, barring a few exceptions. In order for paracellular transport to occur at the BBB, a substance must have a very low molecular weight and be hydrophilic (Kadry et al., 2020). This is in contrast to substances that can diffuse transcellularly, which must be hydrophobic (Zhao et al., 2015). BBB TJs are composed of claudins (primarily 3, 5, and 12), occludin, zonula occludens (ZOs), and junctional adhesion molecules (JAMs) (Luissint et al., 2012). Claudins are transmembrane proteins that are anchored in the cytoplasm by ZOs and are largely responsible for the strict regulation of BBB permeability (Yamazaki and Kanekiyo, 2017). β-catenin accumulation in the cytoplasm of BMECs allows for its translocation to the nucleus, where it drives the expression of TJ proteins such as claudin-3. This pathway is reliant on Wnt signaling, which inhibits the sequestration of β-catenin in the cytoplasm (Luissint et al., 2012; Liebner et al., 2008). ZOs interact with intracellular domains of JAMs and occludin to anchor these transmembrane TJ structural proteins to the actin cytoskeleton within BMECs (Luissint et al., 2012; Yamazaki and Kanekiyo, 2017). AJs, which are composed of cadherins and catenins that aid in cell-cell adhesion, support the impenetrable nature of the BBB (Kadry et al., 2020; Stamatovic et al., 2016). Another aspect of vascular physiology that supports the integrity of the BBB is sheer stress, or the force created by the endogenous flow rate of the bloodstream (Cucullo et al., 2011). Indeed, one study illustrates how sheer stress contributes to barrier morphology and function using an *in vitro* model of the BBB and transendothelial electrical resistance (TEER) measurement. The authors demonstrate that transcripts for various TJ and AJ proteins are expressed at levels two to nearly six times higher in BMECs exposed to normal physiological flow rates compared to those exposed to no flow (Cucullo et al., 2011). This indicates a crucial role of the blood supply itself as well as pericytes and astrocytes that contribute to pulsation and flow of the NVU (Kadry et al., 2020).

The basement membrane (also referred to as the basal lamina), although often overlooked in BBB studies, plays a significant role in the structure and maintenance of the BBB (Luissint et al., 2012; Thomsen et al., 2017). The basement membrane is composed of a 50–100 nm thick network of extracellular matrix proteins (primarily collagen IV, laminin, nidogen, and perlecan) surrounding BMECs (Luissint et al., 2012; Thomsen et al., 2017; Xu et al., 2019). Both BMECs and the brain parenchyma have basement membranes, which are separated by pericytes (Xu et al., 2019). Pericytes are capable of direct contact with BMECs through N-cadherin and connexin (Kadry et al., 2020). They provide integral structural support through their morphology (i.e., wrapping around BMECs and their associated basement membranes) and by secreting laminin and other basement membrane proteins, especially during development (Thomsen et al., 2017). Surrounding both basement membranes and pericytes to form an “outer layer” of the BBB are astrocytic endfeet. Astrocytes are the most abundant cell type of the CNS (Kadry et al., 2020) and perform a wide range of functions. Like pericytes, astrocytes are indispensable in BBB regulation and formation during development, during which they release crucial growth factors that contribute to TJ formation (Cabezas et al., 2014). Astrocytes also participate in neurovascular coupling, in which they act as a relay for nutrients and other physiological necessities between neurons and BMECs (Lia et al., 2023). Further, astrocytic endfeet at the BBB aid in metabolic homeostasis and fluidic movement through expression of SLCs and aquaporins, respectively (Kadry et al., 2020; Thrane et al., 2011). Together, the individual components of the NVU create a dynamic structure uniquely equipped to meet the high energy and nutritional demands of the brain while maintaining a stringent vetting system against harmful invaders.

### 3.2 Circadian regulation of efflux transport at the BBB

BMECs are the brain parenchyma’s first line of defense against potentially harmful substances (Kadry et al., 2020). There are various mechanisms directly controlling BBB permeability, and several of these are influenced by circadian oscillations (Cuddapah et al., 2019; Schurhoff and Toborek, 2023). Core clock machinery alters the expression of transport proteins expressed by BMECs (Schurhoff and Toborek, 2023; Pulido et al., 2020). This has been demonstrated using rhodamine 123 (rh123), a small (380.8 Da) fluorescent binding substrate for Pgp. Indeed, the concentration of rh123 in brain tissue is increased when cortical neurons are activated compared to when they are silent in pathologically naïve mice. Furthermore, *Abcb1a* (Pgp), *Abcg2* (BCRP), *Abcc4* (MRP4), *Abca3*, and *Abcd4* (efflux transporters at the mouse BBB) transcripts were all downregulated in BMECs when cortical neurons were activated, and upregulated when they were silenced (Pulido et al., 2020). The peak of rh123 relative fluorescence was observed at the onset of the inactive phase, with the trough at the onset of the active phase. The peak of *Abcb1a* mRNA also corresponds with the onset of activity. However, A*bcb1* mRNA levels decline throughout the active phase and increase during the inactive phase (Pulido et al., 2020). Mechanistically, *Bmal1* has been demonstrated to regulate oscillations in Pgp activity, as *Bmal1* knockout mice exhibit reduced *abcb1a* mRNA levels and a loss of the rhythmic efflux of Rh123 (Pulido et al., 2020). Corroborating data demonstrate a trough of Pgp efflux activity shortly following the onset of activity in rats using positron emission tomography imaging of a radiolabeled tracer (Savolainen et al., 2016). These results suggest time-of-day differences in efflux activity at the BBB (peaking during the inactive phase), and that this occurrence is potentially driven by oscillations in expression of efflux transport proteins in ECs (Pulido et al., 2020; Savolainen et al., 2016).

Conversely, studies have demonstrated that Pgp efflux activity is higher during the active phase than during the inactive phase in multiple species. One study utilizing high-performance liquid chromatography to quantify the Pgp substrate quinidine following intracerebral microdialysis illustrates that Pgp efflux activity peaks during the active phase in rats (Kervezee et al., 2014). More recently, this observation was further supported when researchers determined that higher ratios of brain:blood rhodamine B (a metabolite of rh123 as well as a Pgp substrate) were retained in *Drosophila* when the molecule was injected during the inactive phase (Zhang et al., 2018). It is worth noting that rats are nocturnal vertebrates and *Drosophila* are diurnal invertebrates. Further, daily oscillations in efflux transport at the BBB have also been attributed to second messenger modulation of efflux proteins at the *Drosophila* BBB (Zhang et al., 2018)*.* Here, the authors report antiphase rhythms in Ca^2+^ (a Pgp inhibitor) and Mg^2+^ (a Pgp activity driver) concentrations, with peak Pgp activity occurring during the active phase. The source of these ions is thought to be connected to gap junction (GJ) proteins, as evidenced by the aberrant concentrations of Mg^2+^ and Ca^2+^ in flies with inhibited innexin function in sub-perineural glial (SPG) cells (Zhang et al., 2018). The role of Mg^2+^ has also been demonstrated as an important regulator of BBB efflux in mammals. In a study using human microvascular endothelial cells to understand mechanisms of circadian regulation in the BBB, *Bmal1* was reported to directly drive the expression of Mg^2+^ transporter *TRPM7*. When *TRPM7* was knocked down, intracellular Mg^2+^ concentrations were reduced and the rhythmic oscillations of both Mg^2+^ and rhodamine B efflux were abrogated (Zhang et al., 2021). Together, these results suggest that circadian clock genes may regulate BBB efflux activity via the transcriptional regulation of Mg^2+^.

Inconsistencies within the field may be attributed to variations among species; of the aforementioned *in vivo* studies, only the one conducted in *Drosophila* represented a diurnal species. Although widely accepted as a useful *in vivo* model of the BBB, *Drosophila* are invertebrates with open circulatory systems containing hemolymph rather than blood. Furthermore, their BBBs are composed of a layer of perineural glial (PG) cells directly exposed to hemolymph, and a layer of SPG cells surrounding the abluminal surface of PG cells (Cuddapah et al., 2019). In *Drosophila*, gap and septate junctions separate cells within the BBB. This is in contrast to the mammalian BBB that is primarily regulated by TJs, which are more selective. Despite these differences, the *Drosophila* BBB shares structural similarity with that of mammals. Both express efflux transporters and are comprised of layers of different cell types in communication with each other to serve similar functions (Cuddapah et al., 2019). However, potential antiphase activity cycles (i.e., diurnal *versus* nocturnal) between species in the studies discussed could explain the differences reported between time at which efflux activity is highest at the BBB (Yan et al., 2020). This is not always the case, however, as biological rhythms between diurnal and nocturnal species are not always antiphase (Yan et al., 2020). More research is needed to properly compare and contrast clock gene oscillations within the components of diurnal and nocturnal BBBs, as well as the mechanisms by which they determine time of peak permeability.

Although contrasts in circadian control of the differing structural elements of *Drosophila* and mammalian BBBs could account for different times of increased permeability, the same cannot be concluded about conflicting data between mice and rats (Pulido et al., 2020; Kervezee et al., 2014), both nocturnal species. Further research is required to reach a consensus within the field. However, it is also possible that variations in research approaches may account for these differences. Currently, there are several methods [reviewed elsewhere (Sloan et al., 2012; Saunders et al., 2015)] used by researchers to study BBB permeability. Considering each method presents its own unique advantages and challenges, a “gold standard” in methodology has yet to be adopted. Lastly, the vast majority of studies on circadian control of efflux transport at the BBB are focused on Pgp. To fully elucidate how daily oscillations affect efflux transport, other transporters, such as BCRP and MRPs, must be studied. Additionally, future research should investigate how effects observed at the rodent BBB translate to humans, as different species express varying levels of each transporter (i.e., rodents express more Pgp at the BBB than humans, while humans express more BCRP than rodents) (Morris et al., 2017).

### 3.3 Circadian regulation of paracellular transport at the BBB

Research on circadian regulation of paracellular permeability of the BBB is limited, especially when compared to other biological barriers. Studies exploring oscillations of ECs and TJs have primarily focused on tissues such as the gastrointestinal tract, liver, and retina. This is likely due to the specialized structure of TJs in the BBB (Kniesel and Wolburg, 2000), which sets them apart from similar peripheral barriers (discussed above). Given the protective nature of TJ proteins, circadian regulation of their expression could modulate time-of-day differences of paracellular permeability at the BBB. Indeed, claudin-5 (*Cldn5*; the primary claudin expressed at BBB TJs) transcript levels oscillate in a circadian manner (i.e., in constant conditions) in the cerebellum, hypothalamus, brainstem, and retina in C57BL/6J mice (Zhang et al., 2021). *Cldn5* transcript levels rise throughout the active phase, peak around the onset of inactivity, and decline throughout the inactive phase (Hudson et al., 2019). In the retina, these rhythms are ablated when *Bmal1* is silenced in vascular ECs. The same results have been observed *in vitro* in serum-shocked BALB/c brain endothelial (bEnd.3) cells. Similarly, silencing *Bmal1* in these cells results in decreased TEER readings (Hudson et al., 2019), indicating an increase in overall permeability. Mechanistically, knocking down *Bmal1* disrupts Wnt signaling, which prevents the stabilization of β-catenin (Guo et al., 2012). As β-catenin is necessary to drive the transcription of TJ genes, this serves as a potential mechanism by which circadian clock genes regulate paracellular transport dynamics at the BBB. Finally, sleep disruption is also capable of disturbing paracellular transport in the BBB. Indeed, chronic sleep restriction increases sodium fluorescein (a 376 Da fluorescent molecule that crosses the BBB primarily through TJs) quantities within the hippocampus (Hurtado-Alvarado et al., 2017). Furthermore, transcript and protein levels of occludin, claudins 1 and 5, and ZO-1 and -2 are significantly downregulated in C57BL/6 mice following 6 days of chronic sleep disruption (He et al., 2014). In both studies, the expression of TJ markers is rescued with sleep recovery (Hurtado-Alvarado et al., 2017; He et al., 2014).

### 3.4 Other rhythms at the BBB

Because most studies on rhythms in BBB function are focused on EC-specific mechanisms, the study of oscillations in the various functions of supporting cells (i.e., astrocytes and pericytes) is an almost completely untapped area of research. Astrocytes, which comprise a dense population of the SCN, display rhythms that are crucial to the function of the central pacemaker, especially during the circadian night (Brancaccio et al., 2017; Tso et al., 2017). Maintenance and regulation of the SCN ultimately aid in circadian regulation of the BBB. However, rhythms in astrocytic endfeet, as they specifically relate to BBB permeability, are vastly understudied. Pericytes, an important component of the vascular basement membrane, also play a crucial role in sustaining the integrity of the BBB. While daily rhythms pertaining to essential functions in these cells have not been well characterized, hindrance of the molecular clock in both the central and peripheral nervous systems (using *Bmal1*_nestin_^−/−^ mice) resulted in significant loss of pericyte coverage of the BBB in the adult murine cortex (Nakazato et al., 2017). The authors demonstrated that this was due to dysfunction in nestin^+^ pericytes, rather than an altogether drop in the number of pericytes. These data suggest that the molecular clock contributes to maintenance of the BBB by upkeeping pericyte coverage. There is currently little evidence to suggest that other types of transport at the BBB (e.g., passive diffusion and SLCs) have daily rhythms in their functions. Further investigation is required to elucidate other potential mechanisms of circadian control at the BBB.

## 4 Circadian rhythms and the blood-cerebrospinal fluid barrier

### 4.1 Overview of the blood-cerebrospinal fluid barrier

**FIGURE 3:**
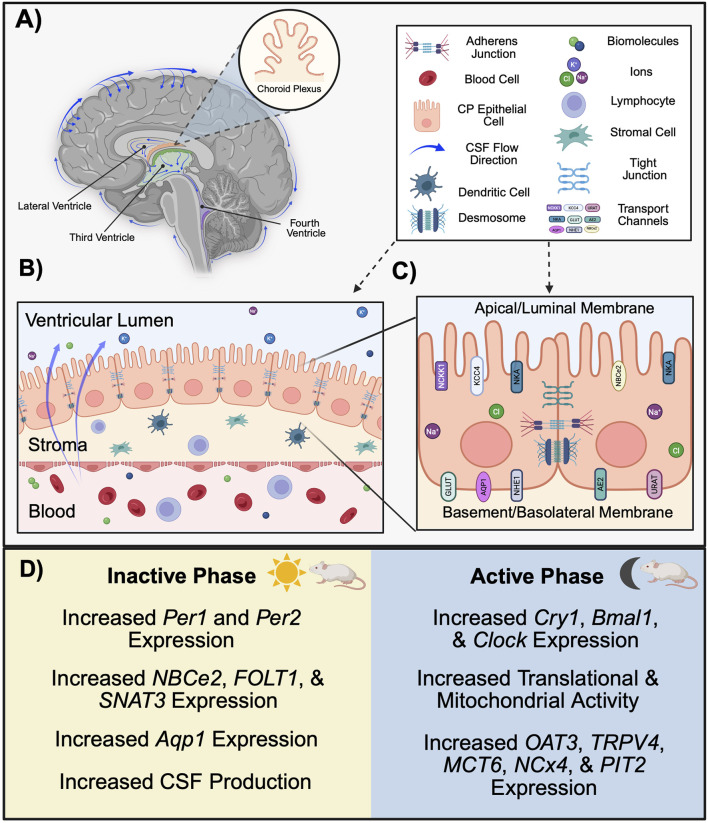
Function and structure of the BCSFB in rodents. **(A)** The choroid plexi, located in the lateral (orange), third (green), and fourth (blue) ventricles, form the BCSFB and generate CSF. CSF circulates throughout the cerebral ventricles and surrounds the brain. **(B)** A monolayer of ciliated cuboidal epithelial cells forms the CPE. The apical (CSF-facing) membrane borders the lumen of the ventricle, whereas the basolateral/basement (blood-facing) membrane borders fenestrated capillaries within the stroma. **(C)** Transport channels located at CPE membranes allow for the passive diffusion of lipid soluble molecules and water. Paracellular transport of the BCSFB is regulated by several junction complexes located between CP epithelia. **(D)** In nocturnal rodents, there are circadian-dependent differences in structure and function of the BCSFB across the inactive and active phase. Clock genes *Per1* and *Per2* are most highly expressed in the CP during the inactive phase, whereas the expression of *Cry1*, *Bmal1*, and *Clock* in the CP is greatest during the active phase. The expression of several efflux transporters varies across the circadian day. Functionally, CSF characteristics change according to time-of-day, with production volumes peaking during the inactive phase. (Aqp1, aquaporin 1; BCSFB, blood-cerebrospinal fluid barrier; Bmal1, brain and muscle ARNT-like protein 1; Clock, circadian locomotor output cycle kaput; CP, choroid plexus; CPE, choroid plexus epithelium; Cry1, cryptochrome 1; CSF, cerebrospinal fluid; Per1, period 1; Per2, period 2. Schematic created with BioRender.).

The blood-cerebrospinal fluid barrier (BCSFB) works in coordination with the BBB to regulate the transport of molecules in extracellular fluid between the periphery and CNS, including ions, amino acids, neurotransmitters, hormones, metabolic waste, and xenobiotic substances (Redzic, 2011). A primary function of the BCSFB is the production of cerebrospinal fluid (CSF), which circulates in a pulsatile manner throughout the cerebral ventricles, subarachnoid spaces (SAS), cisterns, and spinal column (Brinker et al., 2014). The BCSFB is also a key regulator of CSF composition, which is important as it is capable of altering neuronal activity (Brown et al., 2004). CSF composition generally includes vitamins, electrolytes, glucose, and neurotransmitters, as well as low levels of immunoglobulins and proteins (Sakka et al., 2011; Wichmann et al., 2022; Spector et al., 2015). Compared to blood plasma, CSF contains lower concentrations of protein, glucose, and some ions (Czarniak et al., 2023). The primary site of CSF production occurs at the four choroid plexi (CP; Figure 3A), including the hindbrain choroid plexus, two telencephalic CPs, and diencephalic CP, which are located in the fourth, lateral, and third ventricles, respectively (Lun et al., 2015). Despite their distinct classifications, the diencephalic CP fuses to form a continuous structure with the telencephalic CP throughout development (Lun et al., 2015). Whereas the CP is considered to be the greatest producer of CSF (Damkier et al., 2013), the cerebral endothelium and ventricular ependyma have been proposed as potential sites of CSF genesis (Brinker et al., 2014; MacAulay et al., 2022).

The CP is the primary site associated with BCSFB function (Solár et al., 2020). However, the arachnoid membrane and its villi, which envelop the outermost layer of the brain, also serve as a partition between the blood and CNS. There, arachnoid fibroblast-like cells separate fenestrated capillaries in the dura from the CSF in the SAS (Freret and Boire, 2024). As such, some researchers regard the arachnoid-CSF interface as an independent CNS barrier (Freret and Boire, 2024). However, given that the majority of studies examining blood-CSF interfaces examine the CP, it will be the primary focus reviewed here. The fern-like structure of the CP consists of a monolayer of cuboidal epithelial cells known as the choroid plexus epithelium (CPE; Figure 3B), which resides adjacent to, and is continuous with, ventricular ependymal cells (Brissette and Watt, 2022). Importantly, the CP serves as the selective physical barrier preventing peripheral components from entering into CSF (Ayub et al., 2021). Whereas the apical (interchangeably referred to as the luminal membrane or CSF-facing) side of these cells protrude into the ventricular lumen and maintain a dense coat of microvilli, the basement (basal, basolateral, or abluminal) membrane borders connective tissue on the blood-facing side (Figure 3C) (Redzic, 2011; Redzic and Segal, 2004). The basement membrane contains several type IV collagen molecules that allow for the renal-like selective permeability of substances across CP epithelia (Kaur et al., 2016; Urabe et al., 2002). The receptors of several neuropeptides, neurotrophins, and growth factors, including VIP, neuropeptide Y, melatonin, histamine, AVP, insulin-like growth factors, vascular endothelial growth factor, glucocorticoids, prolactin, leptin, and orexin, are located in the epithelial membrane (Solár et al., 2020; Nilsson et al., 1992; Santos et al., 2017). Thus, the BCSFB also has key roles in immune surveillance and neurogenesis (Ayub et al., 2021; Santos et al., 2017).

Located below the CPE are highly fenestrated capillaries within the stroma (Solár et al., 2020). At the CPE, junction complexes regulate the active transport of water-soluble molecules across the BCSFB (Solár et al., 2020). Nearest the basolateral membrane of the CPE are AJs, which bind to the cell’s actin cytoskeleton via a cadherin-catenin complex (Castro Dias et al., 2019). Similarly, nectin-anchored GJ complexes located inferior to AJs aid in cell-to-cell adhesiveness (Solár et al., 2020; Castro Dias et al., 2019). Finally, a string of TJs located near the apical membrane of the cell function to restrict or permit the paracellular movement of substances across the BCSFB. To understand the complexity of this barrier and its function, it is important to address qualities that differentiate the BCSFB from other barriers. Of particular relevance, several factors pertaining to transport activity are unique to the BCSFB.

Compared to the BBB, the BCSFB demonstrates lower TEER values, suggesting a greater paracellular permeability (Redzic, 2011). Indeed, the transport of large molecules is more frequent and efficient at the BCSFB (Sanchez-Covarrubias et al., 2014). Morphologically, it is important to consider that transport occurring at the BBB occurs solely via BMECs; although CP capillaries are flanked by endothelial cells, the epithelial cells of the CP are responsible for forming the physical barrier of the BCSFB. Differences in paracellular transport between barriers arise not only from structural differences, but also the barrier-specific expression, location, and physiology of its transport molecules (Morris et al., 2017). For example, claudins differ in quantity and subtype expression between barriers, which likely contributes to the relative “leakiness” of the BCSFB compared to the BBB (Redzic, 2011). Claudins 1-3 are the primary claudins expressed in the CPE, but are lowly expressed in ECs (Steinemann et al., 2016; Mineiro et al., 2023). Whereas claudin-1 and -3 form seals within TJs, claudin-2 aids in the paracellular transport of cations, functionally resulting in an increase in water permeability across the CPE (Mineiro et al., 2023). Further, histological studies demonstrate that, despite its role as a vital size-selective modulator at the BBB, claudin-5 is absent within the CPE (Steinemann et al., 2016). In addition to paracellular mechanisms, components of efflux transport also vary between barriers. Among the ABC transporters identified within the CP, Pgp and MRP are the most highly expressed (Solár et al., 2020). Whereas Pgp is expressed both luminally and abluminally in the human BBB, it is only apically present in the CPE (Morris et al., 2017). Species-specific differences also exist, as Pgp expression is limited to the luminal surface of the BBB in rodents (Morris et al., 2017).

The polarity of the CP membrane is necessary for creating concentration gradients that confer efflux transporter activity and CSF production (Jessen et al., 2015). Electrochemical gradients are determined by the location of transmembrane channels, which differ in CP epithelial cells compared to other barriers. Na^+^-K^+^-ATPase (NKA) and Na^+^-K^+^-2Cl^−^cotransporter 1 (NKCC1) are stationed on the apical side of the CPE, which contradicts the basal location observed of the same transporters in other epithelial barriers (Damkier et al., 2013). Functionally, transporters including Na^+^/H^+^ exchanger 1 (NHE1), Na^+^- HCO^3-^ cotransporter (NCBE), and Cl-/HCO^3-^ exchanger (AE2) at the basolateral membrane work in conjunction with Na^+^-HCO^3−^ cotransporter (NCBe2), K^+^-Cl^−^ cotransporter KCC4, and anion channels at the luminal surface to create an ionic flux towards the ventricles (Solár et al., 2020; Jessen et al., 2015). The transport of Na^+^, Cl^−^, and HCO^3-^ creates an osmotic gradient, allowing for the passage of water from the blood to the lumen (Jessen et al., 2015). Although these serve as only a few examples of subcellular differences between the barriers, they suggest that factors influencing the operation and greater objective of each structure are barrier-specific. Therefore, although commonly observed throughout CNS barrier literature, the authors make the argument that it is not appropriate to characterize the two systems together when discussing mechanisms of barrier function. Further efforts should be made to identify other molecular variations between the two, which likely result in functional differences and may be subject to varying levels of circadian control.

### 4.2 Circadian regulation of the CP and CSF dynamics

Key homeostatic functions of the BCSFB are firmly regulated via circadian control (Figure 3D), and this is in part due to an autonomously-driven circadian rhythm within the CP. Several clock genes are rhythmically expressed within the CPE, including *Cry1*, *Cry2*, *Per1-3*, *Clock,* and *Bmal1*, with oscillations occurring approximately every 24 h (Quintela et al., 2021)*.* Intriguingly, one study utilizing bioluminescence to quantify the PER2 protein in the brain demonstrates that the CP exhibits a more robust circadian rhythm compared to other brain regions where *Per2* is expressed, including the SCN (Myung et al., 2018a). RNA sequencing data provide further evidence of CP rhythmicity, as the expression of *Per1* and *Per2* peaks during the inactive phase whereas *Clock*, *Bmal1,* and *Cry1* peak during the active phase for rats (Edelbo et al., 2023). Additionally, rodent studies demonstrate that the expression of clock genes within the CP occurs in a sex-dependent manner (Quintela et al., 2015). This phenomenon is likely hormone-dependent, as evidenced by the expression of sex hormone receptors within the CP, and may potentially underlie the observation of greater CSF volumes in female mice compared to males (Quintela et al., 2015; Liu et al., 2020). In another sequencing study, genes associated with translation and mitochondrial activity were most highly expressed during the dark phase (Fame et al., 2023). Together, these findings provide insight for time-of-day variations in BCSFB activity, such the regulation of CSF dynamics.

CSF volumes substantially increase during the dark phase in both rodents and humans, providing evidence against previous claims attributing sleep as the primary determinant of CSF production (Steffensen et al., 2023; Edelbo et al., 2023). In addition to changes in CSF volume, studies examining time-of-day effects on CSF composition demonstrate fluctuations in metabolite concentrations. Notably, a study recently performed in rats demonstrated that 22% of CSF metabolites differ in quantity between the day (*zeitgeber* time [ZT] 8) and night (ZT 20) (Edelbo et al., 2023). These diurnal fluctuations in CSF volume and composition are likely the result of circadian-dependent transport mechanisms at the BCSFB.

The SCN has recently been established as a critical regulator of CP rhythms. In a study aimed to identify the influence of the SCN on the CP, Sládek and coauthors demonstrate that surgically lesioning the SCN in adult mice substantially reduces CP rhythmicity (to the extent of clock gene deletion) both *in vivo* and *ex vivo* (Sládek et al., 2024). Robust CP rhythmicity is rescued in SCN-lesioned mice administered dexamethasone, suggesting that glucocorticoids may indeed serve as one mechanism by which the SCN drives BCSFB regulation (Sládek et al., 2024). In addition to establishing a supportive role of the SCN for the CP clock, the authors performed temporally resolved transcriptomic sampling to identify the circadian-dependent expression of genes important for CP homeostasis. Of those not identified as clock transcripts, genes exhibiting circadian rhythmicity are involved in the endoplasmic reticulum stress response, CPE immune defense, CSF production and composition, and metabolite clearance (Sládek et al., 2024). In sum, results from this study provide novel insights into the circadian-dependent regulation of several BCSFB functions in male mice.

Finally, it is important to address the evidence supporting a significant role of the CP in circadian regulation. The relationship between circadian rhythms and the CP extends beyond that of BCSFB regulation, with evidence suggesting that the CP is capable of influencing the global circadian clock. Indeed, data from a recent *in vitro* study suggest that the pace of the SCN is accelerated when co-cultured with CP explants (Myung et al., 2018b). A reciprocal relationship between the two structures has also been demonstrated *in vivo* (Myung et al., 2018a). While the exact mechanism by which the CP may govern the SCN has not been definitively established, diffusible factors within the CSF have been identified as a potential culprit (Myung et al., 2018a). This raises the possibility that the BCSFB serves as an indirect pathway via which peripheral signals could influence circadian regulation in a bottom-up manner.

### 4.3 Circadian regulation of efflux and influx transport at the BCSFB

NKCC1, located on the apical side of the CPE, is under circadian regulation and plays a key role in regulating ion transport and concentrations. As such, the circadian-dependent expression of NKCC1 likely contributes to time-of-day differences in CSF production (Quintela et al., 2021). Although several other efflux transporters are present within the BCSFB, how these transporters may be regulated by circadian mechanisms and the extent to which they result in functional differences at the BCSFB has not been well established. However, results from a study examining daily oscillations in ABC and SLC transporters within the CP reported both circadian- and sex-dependent differences in expression (Furtado et al., 2022). To test whether variations in expression resulted in functional differences, the authors measured the transport of methotrexate, a substrate of several ABC transporters, and found that concentrations also varied in a circadian manner (Furtado et al., 2022). Other work suggests that organic anion transporter 1 (OATP1; SLC22A6), transient receptor potential vanilloid 4 ion channel (TRPV4) and several members of the solute transporter family including monocarboxylate transporter 6 (MCT6; SLC16A2), sodium/calcium exchanger NCX4 (SLC24A4), and phosphate transporter 2 PIT2 (SLC20A2) are upregulated during the dark phase (Edelbo et al., 2023). In contrast, folate transporter 1 FOLT1 (SLC19A1), the glutamine regulating sodium-coupled neutral amino acid transporter 3 SNAT3 (SLC38a3), and sodium bicarbonate cotransporter 2 (NBCe2; SLC4A5) are upregulated during the light phase in the CPE (Edelbo et al., 2023). The expression of ABC and SLC transporters are regulated by nuclear receptors, which are regulated by core clock genes, providing a potential mechanism by which core clock genes influence efflux activity at the BCSFB (Quintela et al., 2021).

Finally, aquaporins (primarily, aquaporin 1 [AQP1]) are mostly expressed on the apical membrane of the CPE and increase the efficiency of fluid transport across the epithelium (Damkier et al., 2013). Provided its role in water channeling, AQP1 expression has been investigated as a potential mechanism underlying oscillatory changes in CSF volume across the circadian cycle. Although one study investigating the expression of AQP1 reports an absence of rhythmicity (Yamaguchi et al., 2020), results from other studies demonstrate robust day-night differences in aquaporin expression. Indeed, a recent transcriptomic study demonstrates that aquaporin is substantially elevated during the light phase in rats, with AQP1 transcripts alone contributing to 28% of the top 20 differentially expressed genes within the CP (Edelbo et al., 2023).

### 4.4 Circadian regulation of paracellular transport at the BCSFB

Between epithelial cells, GJs maintained by connexin-43 (Cx43) allow for the synchronization of individual cellular circadian rhythms, with the period of the organ-level circadian rhythm being mediated by the strength of these connections. The strongest evidence supporting this role of GJs comes from a study in which administration of the Cx43GJ blocker was associated with an extended length of the CP circadian period (Myung et al., 2018a). Meanwhile, evidence suggests that in addition to influencing the circadian cycle, the regulation of TJs within the CP is also dependent on circadian processes. A study assessing photoperiod length on CP function in ewe determined that ZO-1, ZO-2, cadherin, and afadin expression was downregulated under long photoperiods (Lagaraine et al., 2011). This outcome was functionally associated with decreased CSF turnover and greater concentrations of proteins within CSF, indicating an increase in CP paracellular permeability during periods of extended light exposure (Lagaraine et al., 2011).

### 4.5 Other rhythms at the BCSFB

Both innate and adaptive immune cells have been identified within the BCSFB (Lazarevic et al., 2023). Despite systemic inflammation resulting in the large recruitment of immune factors at the CP, which are capable of altering circadian processes (Carare et al., 2008; Zielinski and Gibbons, 2022), results from a recent study demonstrate CP clock genes are unaffected following an LPS challenge, suggesting that the BCSFB circadian clock is highly resistant to neuroinflammation (Drapšin et al., 2024). In contrast, the same study demonstrates that CP rhythmicity is greatly sensitive to circadian disruption, with evidence supporting a role for glucocorticoids in the dampening effect rather than central inflammation (Drapšin et al., 2024). This is further supported by a study performed in sheep that revealed immune factors including toll-like receptor 2 (TLR2), toll-like receptor 4 (TLR4), and interleukin 1 beta (IL-1β) are differentially expressed during long and short days in response to an LPS challenge (Skipor et al., 2011). Indeed, these factors, which initiate the synthesis of pro-inflammatory cytokines, are substantially upregulated on short days compared to long days (Skipor et al., 2011). Together, these data suggest that photoperiod may be an important regulator of immune surveillance at the BCSFB.

## 5 Circadian rhythms and the glymphatic system

### 5.1 Overview of the glymphatic fluid system

**FIGURE 4:**
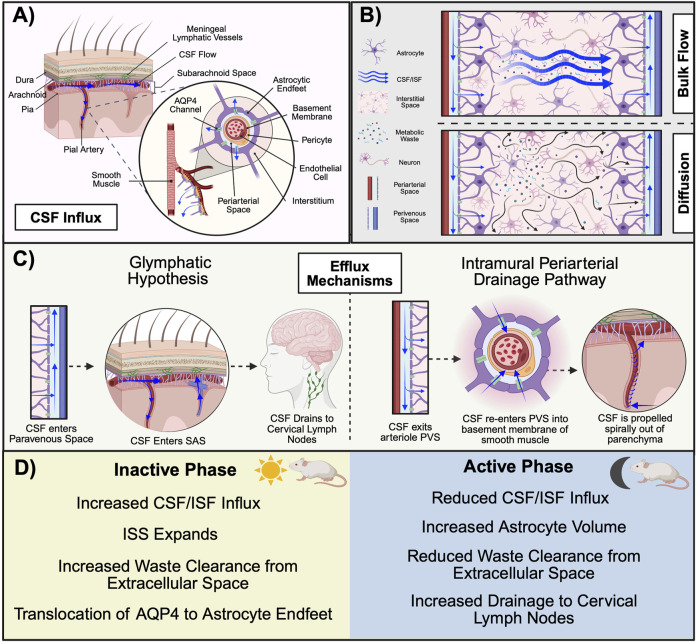
Mechanisms of glymphatic system function in rodents. **(A)** CSF in the SAS descends into the PVS along the pial artery. The PVS is flanked by a wall of astrocytic endfeet that form a cylindrical enclosure around the vascular system. CSF in the PVS is propelled by AQP4 channels on the astrocytic endfeet into the interstitium where **(B)** glymphatic clearance of metabolic waste occurs via either the convective bulk flow of CSF/ISF or diffusion from areas of high concentration to low **(C)** Proposed mechanisms of primary glymphatic efflux include the glymphatic hypothesis (left), in which CSF enters into the PVS of the vein and drains to the cervical lymphatics, and the IPAD (right), in which CSF is expelled from the parenchyma via transport along the basement membrane of smooth muscle cells within the arterial wall. **(D)** There are time-of-day differences in glymphatic function across the inactive and active phase in nocturnal rodents. During the inactive phase, AQP4 translocates to astrocyte endfeet to aid in the influx of CSF into the ISS. Astrocyte volume decreases during this time to increase extracellular space, which likely aids in increased solute clearance. Although CSF/ISF influx and glymphatic clearance are increased during the inactive phase, research performed in rats suggests that glymphatic drainage to the cervical lymph nodes may be highest during the active phase. During the active phase, astrocyte volume increases and CSF/ISF flow are reduced. (Abbreviations: AQP4, aquaporin 4; CSF, cerebrospinal fluid; IPAD, intramural periarterial drainage pathway; ISF, interstitial fluid; ISS, interstitial space; PVS, perivascular space. Schematic created with BioRender.).

The relatively recent identification of a lymphatic system within the brain has revolutionized the way researchers approach concepts including neuroinflammation, sleep, and pathology. Named after its constituents “glial” and “lymphatics,” the glymphatic system was first described in 2012 with the identification of a paravascular pathway extending into the parenchyma of mice (Iliff et al., 2012). Rodent studies have since identified a network of lymphatic vessels invading the cranial sinuses (Louveau et al., 2015), and magnetic resonance imaging (MRI) studies in humans have demonstrated that CSF solutes drain into the cervical lymph nodes (Eide et al., 2018). Thus, recent evidence supports the notion that the glymphatic system extends beyond the BCSFB and plays a key role in the clearance of metabolic waste products and solutes from the parenchyma. Given the overlapping descriptions of CSF and interstitial fluid (ISF) dynamics found across CNS literature, for the purpose of this review, the term “glymphatic function” will be used to convey three specific processes: glymphatic influx (the movement of CSF into the interstitium; Figure 4A), glymphatic clearance (the transport of solutes out of the interstitial space; Figure 4B), and glymphatic efflux (the drainage of waste-transporting fluid from the CNS; Figure 4C). Of note, researchers’ understanding of glymphatic influx is currently the most comprehensively described of these processes.

To briefly review the progression of fluid movement throughout the glymphatic system, CSF enters from the SAS and descends into the subpial perivascular space. The perivascular space (PVS), also referred to as the Virchow-Robbin space, can best be thought of as a cylindrical tunnel surrounding neural vasculature. Magnetic resonance imaging has characterized PVS among the lenticulostriate, medullary, and collicular arteries (Rudie et al., 2018). While the arterial endothelium serves as the inner “wall”, the PVS is flanked exteriorly by a continuum of overlapping astrocyte endfeet that serve as a vascular sheath and effectively encompass CSF. Respiration, vasomotion, and hydrostatic pressure facilitate the paravascular movement of CSF from the SAS (Jessen et al., 2015). As the artery descends and narrows into arterioles, the PVS becomes restricted and eventually merges into the basal lamina of cerebral capillaries, where glial aquaporin 4 (AQP4) channels propel CSF into the interstitium (Jessen et al., 2015; Ding et al., 2023). There, CSF intermingles with ISF, marking the first physical exchange between the CSF and glymphatic systems (Jessen et al., 2015).

ISF encompasses all neural cells and makes up approximately 20% of the total fluid volume within the CNS (Jessen et al., 2015). Although ISF is found in the interstitial space, also referred to as the interstitial system (ISS), the primary site of its production is unknown. Whereas the by-product of either cell metabolism or CSF has been postulated to contribute to ISF volume, some evidence suggests a plasma-derived, transcapillary fluid transfer across the BBB (Shetty and Zanirati, 2020; Xiang et al., 2023). ISF encompasses enzymes, glycosaminoglycans, ions, extracellular vesicles, proteoglycans, and glycoproteins such as collagen, fibronectin, and laminin within the extracellular matrix (Shetty and Zanirati, 2020). Thus, ISF plays an important role in providing nutrients to cells, neuronal cross-talk, and immune regulation (Wiig et al., 2012). Due to the pressure-driven influx of CSF, a traditional hypothesis of glymphatic clearance is that solutes are “flushed” out of the ISS in a convective manner (Figure 3B) (Agarwal and Carare, 2021). Supporting this bulk flow concept are findings from a study utilizing contrast-enhanced MRI, in which comparable transport rates are observed for dyes of varying molecular weights (Iliff et al., 2013). For efflux, waste-transporting fluid enters into the paravenous space where it is transported out of the parenchyma back into the SAS (Bacyinski et al., 2017). There, CSF enters into arachnoid granulations (AG) and travels along collagen channels in the meningeal cell cap, where it is then transported across the venous epithelium and drained into the venous sinus (Weller et al., 2018). Evidence for AG-mediated efflux comes from studies performed *in vitro* that demonstrate higher flow rates and hydraulic conductivity for AGs perfused basally to apically when compared to AGs perfused in the opposite direction (Grzybowski et al., 2006).

Aspects of this process, referred to as the glymphatic hypothesis, have been contested since its debut in 2012. Recent studies provide evidence supporting the existence of alternative clearance mechanisms and efflux pathways. For example, rather than bulk flow being the primary mechanism of solute transfer throughout the ISS, it has been suggested that solutes in the tortuous extracellular space are transported via diffusion, moving in a gradient manner from areas of high concentration to low. One argument for this mechanism is that hydraulic resistance at these locations is too high for bulk flow (Abbott et al., 2018). For efflux it has been postulated that rather than exiting the parenchyma via paravascular transport, ISF is evacuated perivascularly. To clarify, this approach, known as the intramural periarterial drainage pathway (IPAD), proposes that ISF within the parenchyma enters into the basement membrane of the cerebral capillaries via AQP4 channels. From there, ISF is transported into the basement membrane of the smooth muscle cells within the arterial wall (the tunica media) and propelled out of the parenchyma via vasomotion (Wang et al., 2023). Arterial involvement is a defining feature of this proposal, as perivascular spaces are not present within the vein (Bacyinski et al., 2017). However, the nature of this barrier in preventing protein transport suggests this is not likely a substantial mechanism for large solute or cell clearance (Agarwal and Carare, 2021). Evidence for this pathway first emerged with the identification of CSF tracers in the tunica media of histological brain sections and has since been supported by *in vivo* imaging studies (Carare et al., 2008; Veluw et al., 2020)*.* However, the results of these studies are contested by Mestre and colleagues who emphasize that periarterial spaces collapse during fixation to displace CSF tracers and the *in vivo* studies exhibit invasive methodologies that confound experimental outcomes (Mestre et al., 2020). Further, two-photon imaging confirms that periarterial spaces collapse upon death (Mestre et al., 2020).

Finally, studies examining the meningeal lymphatic system have further provided new insights into potential efflux pathways. One such pathway involves the movement of CSF across the cribriform plate towards the nasal mucosa, where it drains into the deep cervical lymph nodes in the neck (Proul, 2021). This likely occurs via either perineural transport along olfactory nerve sheaths or by directly entering into lymphatic vessels (made up of leptomeningeal cells) that extend across the cribriform plate and penetrate into the SAS (Proul, 2021). Other studies have demonstrated that lymphatic drainage occurs via the dural/meningeal lymphatic vessels (Aspelund et al., 2015). Despite the lack of consensus regarding glymphatic efflux, it is known that variables including circadian processes, aging, and body position, are all capable of altering the efficiency of this system (Ding et al., 2023).

### 5.2 Circadian regulation of the glymphatic system

Similar to other CNS barriers, glymphatic function is subject to circadian control (Figure 4D). A study quantifying the amount of CSF tracer in the parenchyma across the 24-h cycle revealed that levels of the tracer are 50% greater at ZT 6 relative to ZT 18 in anesthetized mice, suggesting that glymphatic influx is highest during the day and independent of arousal state (Hablit et al., 2020). Importantly, this observation persisted even when mice were housed in constant conditions for 10 days, lending credence to the notion that glymphatic activity is inherently circadian-driven (Hablit et al., 2020). Further, solute clearance during the inactive phase was increased by over 50%. Although these data were derived from mice anesthetized with a ketamine/xylazine cocktail, which has been indicated to alter aspects of glymphatic transport relative to other anesthetic compounds (Hablitz et al., 2019), results were replicated in mice treated with pentobarbital as well as tribromoethanol. Finally, lymphatic drainage of CSF within the SAS displayed a time-of-day pattern opposite that of other glymphatic functions, as fluorescent intensity in the cervical lymph nodes was greatest during the active phase (Hablit et al., 2020). Mechanistically, it has been suggested that circadian regulation of the NVU is a key component of glymphatic efficiency. For example, smooth muscle cell *Bmal1* expression is an important determinant of vascular integrity and function, as its deletion disrupts contractile protein regulation and alters cerebral flow dynamics (Li et al., 2024). Similarly, the localization of AQP4 to astrocytic endfeet is a time-of-day dependent mechanism necessary for adequate CSF/ISF movement (Vizcarra et al., 2024). Taken together, these studies highlight the existence of circadian-driven variations in glymphatic activity. Still, other studies support a role for sleep in mediating glymphatic activity.

### 5.3 The glymphatic system and sleep

Recent evidence suggests that glymphatic activity levels vary according to vigilance state. The ISS expands substantially during sleep, effectively lowering resistance to CSF influx and aiding fluid movement throughout the extracellular space (Xie et al., 2013). This expansion is in part driven by a sleep-mediated variation in astrocyte volume, which is decreased during sleep (Sriram et al., 2024). These decreases are achieved via the osmotic signaling of molecules within the extracellular matrix and are inversely correlated with extracellular space. Moreover, sleep phase is associated with differing levels of glymphatic activity. For example, glymphatic function is positively correlated with the amount of time spent in slow wave or non-rapid eye movement sleep (Hablitz and Nedergaard, 2021). This increase in activity is likely consequential of the sluggish brain oscillations observed during this phase, which promote vasodilation and, subsequently, an increase in CSF influx (Bojarskaite et al., 2023). Electroencephalography measurements captured simultaneously with bold MRI data reveal that the reoxygenation of the brain, resulting from glymphatic clearance, coincides with slow wave electrical signals in a temporally precise manner (Fultz et al., 2019).

Evidence from foundational studies provides further support for a sleep-mediating mechanism. Opposing the finding that circadian rhythms prevail to drive activity under anesthetic conditions, stark differences in glymphatic function have been observed between awake and asleep *versus* anesthetized mice. In a study using two-photon microscopy to assess CSF tracer dynamics, sleeping mice were administered fluorescein isothiocyanate (FITC)-dextran (3 kDa) midday. A second tracer of the same molecular weight (Texas-red dextran, 3 kDa) was administered after the gentle waking of the mouse, which resulted in a 95% reduction in tracer influx (Xie et al., 2013). FITC-dextran tracing was then performed at night in awake mice, which were then provided a ketamine/xylazine mixture to induce sleep prior to the administration of the second tracer. Similar to naturally sleeping mice, tracer influx was increased in anesthetized mice relative to those awake. This observation was concomitant with a higher prevalence of delta activity (Xie et al., 2013), once again indicating that glymphatic operation is pronounced during slow wave sleep. No differences in tracer influx were detected between sleep induced pharmacologically and natural sleep. To address the role of sleep in glymphatic waste clearance, mice were administered either the radiolabeled peptide ^125^I-Aβ1-40 or the non-permeabilizing tracer ^14^C-inulin. For both molecules, clearance rates were doubled in sleeping and anesthetized mice compared to awake mice (Xie et al., 2013). Thus, maintaining a consistent sleep schedule is vital for the proper regulation of this system. MRI studies demonstrate that patients with self-reported chronic sleep disturbance exhibit impaired clearance of CSF tracers (Eide et al., 2022). Similar outcomes were reported in individuals following only a single night of sleep deprivation (Eide et al., 2021; Shokri-Kojori et al., 2018).

The limited specificity offered by current *in vivo* imaging techniques, use of widely varying methodologies in existing literature, and lack of well-defined efflux pathways pose a challenge for researchers attempting to understand the circadian mechanisms underlying glymphatic function. Although the majority of our mechanistic understanding of the glymphatic system comes from rodent and human studies, similar features of glymphatic architecture are found in a variety of species. While this implies that neural waste clearing may be an evolutionarily conserved process among CNS-containing lifeforms, it is important to acknowledge that factors influencing glymphatic function vary between and within species. For example, an imaging study assessing *in vivo* lymphatic drainage of CSF tracers in human subjects demonstrates that intrathecal tracer enhancement peaks in CSF four to 6 hours following infusion for most individuals, with some variation among individuals (Eide et al., 2018). Peak enhancement in both the brain and cervical lymph nodes, however, occurs 24-h following infusion (Eide et al., 2018). The results are particularly remarkable in that they reveal a large deviation from what is observed in rodent studies, which is that tracer enhancement in the brain peaks at 2 hours (Eide et al., 2018; Iliff et al., 2013). Clearly, the mechanisms underlying these differences warrant further investigation. Until then, circadian determinants of glymphatic activity cannot be ruled out as a possible factor contributing to these differences.

## 6 Conclusion and future directions

Circadian rhythms influence many aspects of animal physiology and behavior, including CNS fluids and barriers. Although there are clear associations between CNS barrier and fluid systems and disease, the role of circadian influences in these relationships remains unspecified. Here, we review the structural and functional components of the BBB, BCSFB, and glymphatics, describe the circadian regulation of each, and provide empirically based insights into the potential role of circadian mechanisms on fluid and barrier systems in disease. A comparative summary of key genes and proteins that are rhythmically expressed in the two physical barriers, the BBB and the BCSFB, is displayed in Table 1.

**TABLE 1:** Comparative summary: circadian influences of the BBB and BCSFB in Rodents.

Key gene or protein	Barrier	Species	Peak expression or activity	Barrier-specific functional implications
Core Clock Genes				
*Bmal1*	BBB	Mouse	ZT0; Onset of inactive phase (Zhang et al., 2021)	Regulates Pgp activity (Pulido et al., 2020)
				Deletion reduces efflux activity (Zhang et al., 2021)
				Drives expression of Mg^2+^ transporter *TRPM7* (Zhang et al., 2021)
	BCSFB	Mouse	ZT23; End of active phase (Fame et al., 2023)	Regulates CP rhythmicity (Fame, 2025)
			ZT0, Onset of inactive phase (Sládek et al., 2024)	
		Rat	Peak expression during active phase (Edelbo et al., 2023)	
*Cry1*	BBB	N/A	Unknown	Unknown
	BCSFB	Mouse	Arrhythmic (Sládek et al., 2024)	Unknown
		Rat	Peak expression during active phase (Edelbo et al., 2023)	
*Cry2*	BBB	N/A	Unknown	Unknown
	BCSFB	Mouse	ZT12; Onset of active phase (Sládek et al., 2024)	Contributes to CP rhythmicity (Sládek et al., 2024)
*Per1*	BBB	Rat	ZT12; Onset of active phase (Durgan et al., 2016)	Unknown
	BCSFB	Mouse	ZT12; Onset of active phase (Sládek et al., 2024)	Contributes to CP rhythmicity (Sládek et al., 2024)
		Rat	Upregulated during light phase (Edelbo et al., 2023)	
*Per2*	BBB	Mouse	Onset of active phase (Zhang et al., 2021)	Unknown
		Rat	ZT18; Active phase (Durgan et al., 2016)	
	BCSFB	Mouse	Active phase (Fame et al., 2023)	Contributes to CP rhythmicity (Sládek et al., 2024)
			ZT12; Onset of active phase (Sládek et al., 2024)	
		Rat	Inactive phase (Edelbo et al., 2023)	
Transporter proteins				
Abcb1a (Pgp)	BBB	Mouse	Active phase (Pulido et al., 2020)	Efflux activity (Pulido et al., 2020)
	BCSFB	N/A	Unknown	Unknown
Abcg2 (BCRP)	BBB	N/A	Unknown	Unknown
	BCSFB	Rat	Peak mRNA expression at ZT14 in intact females only (Furtado et al., 2022)	Unknown
Abcc4 (MRP4)	BBB	N/A	Unknown	Unknown
	BCSFB	Rat	Peak mRNA expression at ZT7 in intact males; Peak mRNA expression at ZT14in intact females (Furtado et al., 2022)	CPE membrane transport (Furtado et al., 2022)
Aquaporins	BBB	Rat	Unknown	Unknown
	BCSFB	Mouse	AQP1; Inactive phase (Edelbo et al., 2023)	CSF and ISF homeostasis; *aqp1-aqp4* KO impairs CSF drainage (Trillo-Contreras et al., 2019)
			AQP4; Perivascular polarization during inactive phase (Hablit et al., 2020)	Global aqp4 KO slows CSF influx (Iliff et al., 2012)
		Rat	AQP1; Arrhythmic (Yamaguchi et al., 2020)	No differences in CSF influx in aqp4 KO mice (Hablit et al., 2020)
SLCs	BBB	N/A	Unknown	Unknown
	BCSFB	Rat	NBCe2, SNAT3; Inactive phase (Edelbo et al., 2023)	CSF secretion (Edelbo et al., 2023)
			MCT6, NCX4, OATP1, PIT2, TRPV4; Active phase (Edelbo et al., 2023)	
Claudins	BBB	Mice	Claudin-5; Onset of inactive phase (Hudson et al., 2019)	Fluctuations in paracellular transport activity (Louer et al., 2020)
			Claudin-2; Active phase (Louer et al., 2020)	
	BCSFB	N/A	Unknown	Unknown

In regard to the BBB, studies assessing the role of circadian influence are often limited to EC mechanisms. Specifically, studies assessing time-of-day variations in BBB function have predominantly focused on efflux transport, and specifically, the role of Pgp. Although evidence exists for time-of day differences in the expression of TJ proteins, it has not been established whether there are circadian-driven fluctuations in paracellular transport and permeability. Therefore, future studies of the BBB should investigate circadian influences on additional cell types in the NVU, transport mechanisms, and substrates. In contrast to the BBB, studies of circadian influence on the BCSFB and glymphatic system are still in their infancy.

The possibility that the BCSFB serves as an indirect pathway through which peripheral signals may alter top-down circadian regulation remains a compelling question worthy of investigation, especially considering that the disruption of circadian-regulated processes is observed in many neuropathologies. Within the CP, epithelial cells demonstrate a robust rhythmic expression of core clock genes and photoperiodic regulation of TJs. However, it is unclear whether this rhythmicity is apparent at the arachnoid region of the blood-CSF interface. Further, although transport proteins found within the BBB have also been identified within the CPE, differences in expression level, membrane location, and functionality have provided researchers with only a shallow understanding of these mechanisms within the BCSFB. As for glymphatics, the lack of consensus regarding glymphatic processes and pathways has limited our understanding of the relationships among circadian biology and CNS barriers and fluid systems. Because differing proposals of glymphatic clearance and efflux likely involve varying extents of circadian control, it is important that researchers first aim to adequately elucidate mechanisms of glymphatic function. Studies aimed at distinguishing the roles of circadian rhythms and sleep on glymphatic activity should also be expanded. Finally, discrepancies in the structure and function of CNS barriers and glymphatic activity between species presents a major limitation in understanding these systems. Further studies should be devoted to identifying these discrepancies, and researchers should be cognizant of how inappropriate conclusions drawn from rodent studies may misguide the study and treatment of human pathology.

Despite these gaps in our knowledge, studies investigating the role of circadian influences on CNS barrier function have broad implications for human health. For example, existing studies have greatly contributed to our understanding of chronotherapy, or the practice of timing drug administration to increase efficacy and reduce adverse effects. Thus far, researchers and clinicians have determined how to modulate the timing of therapeutic delivery to improve treatment efficacy for various conditions such as cancer [reviewed in (Kisamore et al., 2023)] and affective disorders [reviewed in (Swanson et al., 2025)]. By understanding how the function and accessibility of CNS barriers fluctuates across the circadian day, additional chronotherapeutic approaches targeting a wider range of health conditions may be identified. Further, as several pathologies are associated with disrupted CNS barrier structure and function, such as neurodegenerative and cerebrovascular diseases (Hergert et al., 2024), research in this area will aid in elucidating mechanisms underlying the cyclic nature of these relationships. Finally, understanding how disrupted circadian rhythms (such as night shiftwork and nighttime light exposure) alter CNS barrier integrity will aid researchers in identifying preventative measures that may be taken to protect both brain and overall health.

In summary, additional research is required to identify unique aspects of barrier- and species-specific function. Understanding time-of-day differences in CNS barriers and the glymphatic system will remain an important topic for researchers to pursue as chronotherapeutic approaches demonstrate efficacy and continue to grow in popularity. However, a comprehensive understanding of these systems in disease-free models is first required to adequately leverage circadian biology and improve health outcomes for individuals affected by disease.

## Glossary

ABC: ATP-Binding Cassette
AE2: Cl-/HCO^3-^ exchanger
AG: Arachnoid Granulation
AJ: Adherens Junction
AQP1: Aquaporin 1
AQP4: Aquaporin 4
AVP: Arginine Vasopressin
BBB: Blood-Brain Barrier
BCRP: Breast Cancer Resistant Protein
BCSFB: Blood-Cerebrospinal Fluid Barrier
BMAL: Brain and Muscle ARNT-Like Protein
BMEC: Brain Microvascular Endothelial Cell
CAMK: Calcium-Dependent Protein Kinase
cAMP: Cyclic Adenosine 3′,5′-Cyclic Monophosphate
Cldn5: Claudin-5
CLOCK: Circadian Locomotor Output Cycle Kaput
CNS: Central Nervous System
CP: Choroid Plexus
CPE: Choroid Plexus Epithelium
CREB: cAMP Response Element CRE-Binding Protein
CRY: Cryptochrome
CSF: Cerebrospinal Fluid
Cx43: Connexin-43
E-Box: Enhancer Box Regulatory Element
FITC: Fluorescein Isothiocyanate
GJ: Gap Junction
IPAD: Intramural Periarterial Drainage Pathway
ISF: Interstitial Fluid
ISS: Interstitial System
JAM: Junctional Adhesion Molecule
MCT: Monocarboxylate Transporter
MRI: Magnetic Resonance Imaging
MRP: Multi-Drug Resistance Protein
NCBE: Na^+^- HCO^3-^ Cotransporter
NCBe2: Na^+^- HCO^3-^ Cotransporter 2
NHE1: Na^+^/H^+^ Exchanger 1
NKA: Na^+^-K^+^-ATPase
NKCC1: Na^+^-K^+^-2Cl^−^ Cotransporter 1
NVU: Neurovascular Unit
OAT: Organic Anion Transporter
PACAP: Pituitary Adenylate Cyclase-Activating Peptide
PER: Period
PG: Perineural Glial
Pgp: P-glycoprotein
PKA: Protein Kinase A
PVS: Perivascular Space
REV-ERBα/NR1D1: Nuclear Receptor Subfamily 1 Group D Member 1
Rh123: Rhodamine 123
RHT: Retinohypothalamic Tract
RORα: Retinoic Acid Receptor-Related Orphan Receptor Alpha
RRE: Rev Response Element
SAS: Subarachnoid Space
SCN: Suprachiasmatic Nucleus
SLC: Solute Carrier
SPG: Sub-Perineural Glial
TEER: Transendothelial Electrical Resistance
TJ: Tight Junction
TRPV4: Transient Receptor Potential Vanilloid 4 Ion Channel
VIP: Vasopressin Intestinal Protein
ZO: Zonula Occluden
ZT: Zeitgeber Time
